# Comparative Evaluation of Extrusion of Apical Debris in Primary Maxillary Anterior Teeth Using Two Different Rotary Systems and Hand Files: An In Vitro Study

**DOI:** 10.3390/children10050898

**Published:** 2023-05-18

**Authors:** Balaji Suresh, Ganesh Jeevanandan, Vignesh Ravindran, Mohammed Mashyakhy, Noura Alessa, Ather Ahmed Syed, Suman Panda, Ali Ahmed Assiry, Prabhadevi C. Maganur, Satish Vishwanathaiah

**Affiliations:** 1 Department of Pediatric and Preventive Dentistry, Saveetha Dental College and Hospital, Saveetha Institute of Medical and Technical Sciences, Saveetha University, Chennai 600077, India; balajimahadevan18@gmail.com (B.S.); drvigneshpedo@gmail.com (V.R.); 2 Restorative Dental Science Department, College of Dentistry, Jazan University, Jazan 45142, Saudi Arabia; 3 Department of Pediatric Dentistry and Orthodontics, Dental College, King Saud University, Riyadh 11545, Saudi Arabia; nalessa@ksu.edu.sa; 4 Department of Preventive Dental Sciences, Division of Pediatric Dentistry, College of Dentistry, Jazan University, Jazan 45152, Saudi Arabia; drather.ahmed@gmail.com (A.A.S.); sumanpanda73@gmail.com (S.P.); prabhadevi.maganur@gmail.com (P.C.M.); drvsatish77@gmail.com (S.V.); 5 Preventive Dental Science Department, Faculty of Dentistry, Najran University, Najran 1988, Saudi Arabia; aaassire@nu.edu.sa

**Keywords:** apical extrusion, pediatric endodontics, primary teeth, rotary systems

## Abstract

Successful outcome of pulp therapy depends on good chemomechanical preparation of the canals. This is completed with the help of various upcoming rotary and hand files. However, during this preparation, there might be an apical extrusion of the debris which may result in postoperative complications. The aim of this study was to evaluate and compare the number of debris apically extruded during canal preparation using two different pediatric rotary file systems and conventional hand file systems in primary teeth. 60 primary maxillary central incisors that were extracted due to trauma or untreated dental caries with no signs of resorption were taken. Canal preparation was executed using three different file systems: Group A: Group A hand K file system, Group B Kedo S Plus, Group C Kedo SG Blue. For each of these files using the Myers and Montgomery model, the pre- and post-weight of the eppendorf tube was assessed to quantify the number of apical debris. The maximum extrusion of apical debris was noticed with the Hand K-file system. The least debris was noticed in the Kedo S Plus file system. Statistical analysis revealed that there were highly significant differences in apical extrusion and debris when comparing hand files and rotary files and also between the two rotary files used. Apical debris collection is an unavoidable outcome of canal instrumentation. Among the file systems compared, rotary files had lesser extrusion when compared to hand files. Among the rotary files, Kedo S plus showed normal extrusion compared to SG Blue.

## 1. Introduction

Primary teeth are fundamentally responsible for the normal growth and development of the alveolar region of the maxilla and the mandible providing a path of eruption for the succedaneous teeth, thereby allowing physiologic remodeling of the jaws. Early loss of deciduous teeth leads to various complications including space loss, an alternated path of eruption of the successor teeth and may also cause alteration in speech [1]. Restorative and endodontic treatments help both primary and permanent dentition by maintaining the integrity of the teeth which are affected by dental caries [2,3,4,5]. A pulpectomy, being the prime choice for treating pulpal pathologies, aims to clean and shape root canals to receive a suitable obturating material which lends a helping hand to retain the deciduous teeth until its physiologic resorption is initiated [5,6,7]. A pulpectomy is a routine pediatric dental procedure that follows the procedural sequence of local anesthesia administration, rubber dam isolation, access cavity preparation, working length determination, chemomechanical preparation, obturation and a full coverage restoration.

Chemomechanical preparation is a crucial sequence essential for the highly successful prognosis of pulpectomy in primary teeth [8,9]. Previously the mechanical preparation of the canals was completed using hand files. With the continuous growth in pediatric endodontics, currently, the canals are prepared using engine-driven endodontic files [10,11,12]. Though rotary files were introduced in the late 1980s after the introduction of NiTi alloy in dentistry, newer and more efficient rotary files have started to flourish in the dental market. Barr et al. was the first published study to suggest the use of NiTi rotary files in primary dentition [13,14,15]. Although the author suggested the use of a rotary file system that has been used in permanent dentition to be used in primary dentition, there were no rotary file systems designed specifically and exclusively for use in primary dentition until 2017. Kedo-S rotary file systems were the first of their kind to be designed for primary teeth which had undergone periodic customisations in the past quinquennium that led to a paradigm shift in pediatric endodontics. The regular endodontic file systems in the market use four to five file sequences to be used in a sequential manner, while the Kedo-S files suggest only two files and the recently introduced version, is a single file system.

The apical extrusion of debris is an unavoidable factor during canal preparation in both manual and rotary file systems. The extruded debris might have irritants that induce periapical inflammation leading to postoperative pain and a possibility to damage the permanent tooth bud [15,16,17,18]. The number of extruded debris depends on multiple factors, among which instrument design and method of instrumentation play a major role [19]. Although various studies suggest hand files lead to greater debris extrusion compared to rotary files, conflicting results still exist with the design and method of instrumentation [20]. With the recent generation of file systems aiming towards simplification of endodontic treatment by manufacturing single file systems [21], there is no published data on the over-preparation of the canal and the design of file systems in relation to the extrusion of apical debris in the primary dentition. The aim of this study was to evaluate and compare the number of debris apically extruded during canal preparation using two different pediatric rotary file systems and conventional hand file systems in primary teeth.

## 2. Materials and Methods

### 2.1. Study Design and Characteristics

This in vitro study was performed on extracted human primary teeth samples. Ethical Approval was obtained from the institutional scientific and research committee (IHEC/SDC/PEDO-2103/22/649). The sample size was calculated using G power analysis (95% Power) from similar research conducted earlier in our institute [22]. The calculated sample size was a total of 60 teeth.

### 2.2. Study Samples

The teeth used in this study were primary maxillary central incisors that were extracted due to trauma or due to untreated dental caries with no signs of physiological or pathological resorption. As the parents were not willing to pulp therapy such teeth were extracted and preserved until the study was performed. Signed consent was obtained from the parents after informing them about the use of the extracted teeth for research purposes. Single-rooted primary maxillary central incisors were taken. The presence of a single canal and a single apical foramen with no additional canal aberrations was verified using buccolingual and mesiodistal radiographs. The teeth samples were excluded when any sign of resorption or presence of more than one canal or calcified canal was noticed. The extracted teeth were randomly assigned to three different groups using a block randomization method with a block size of three. A computer-generated randomization list was used to assign participants to treatment groups within each block. (i.e., 20 teeth to each group).

### 2.3. Teeth Sample Preparation

Extracted teeth were disinfected using formalin 10% solution and stored in distilled water till the study was initiated [23]. Access preparation was completed using no. 6 round diamond bur (Mani Inc., Tochigi, Japan) using a high-speed air-rotor handpiece. Canal patency was checked using a size 10 K-file (Mani. Inc., Tochigi, Japan). The working length for each tooth was measured using a size 15 K-file (Mani. Inc., Tochigi, Japan) until the tip of the file was visible at the apical foramen. The rubber stopper was adjusted to the most stable point at the coronal region. The length was measured using a ruler and the working length determined was kept at 1 mm short of the apical foramen. All the teeth samples were numbered, and the working length was noted for each tooth sample for reference during the biomechanical preparation.

### 2.4. Experimental Model Design

**Figure 1:**
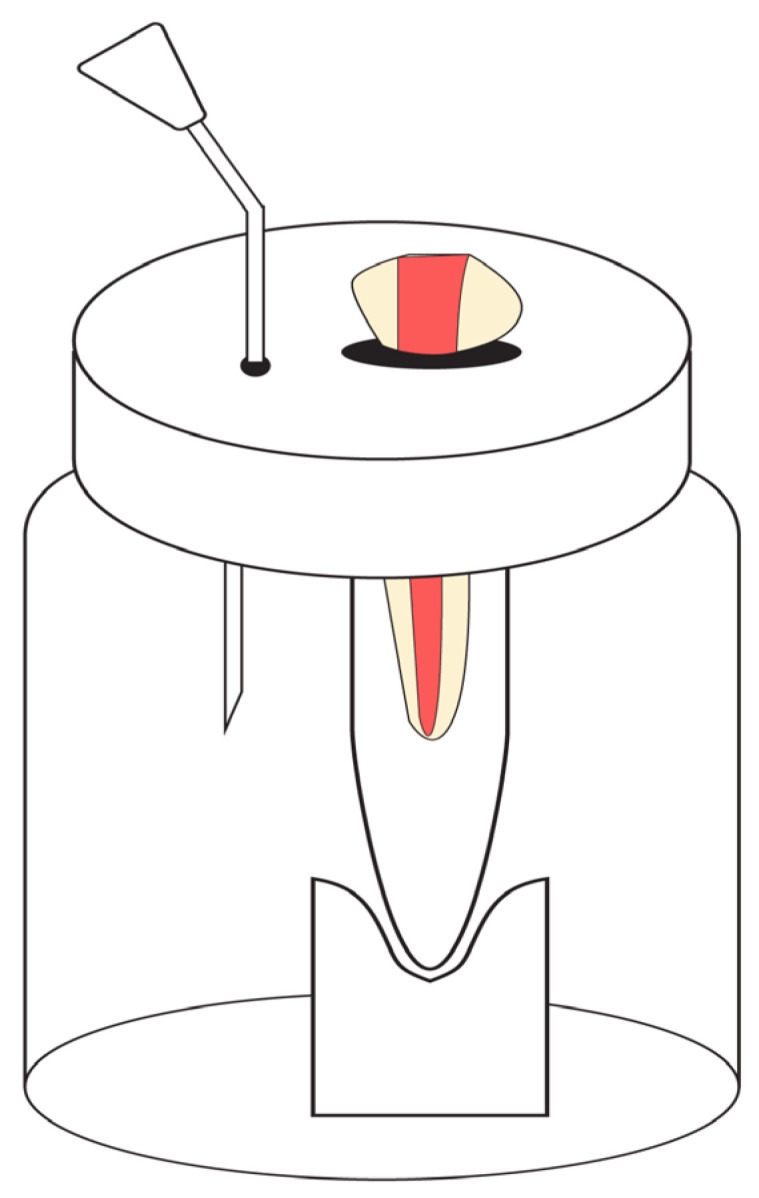
Myers and Montgomery model used in the present study.

The experimental model used for this in vitro study was Myers and Montgomery model [24]. Eppendorf tubes were used to collect the apical debris. Empty Eppendorf tubes were preweighed by the electronic microbalance (Sartorius, Germany Model-CP225D) of 10^−5^ gm sensitivity. The 60 Eppendorf tubes (20 Eppendorf tubes for the 20 teeth per group) were preweighed three consecutive times and the arithmetic mean values were recorded. The teeth were held to a pre-weighed Eppendorf tube using a rubber stopper, and this assembly was fixed to a glass container wrapped using aluminum foil to avoid observer bias by the operator during the instrumentation (Figure 1). Then a 30-gauge (BD microlance, India) needle was used to vent and equalize internal and external pressure within the glass container.

### 2.5. Root Canal Preparation

**Figure 2:**
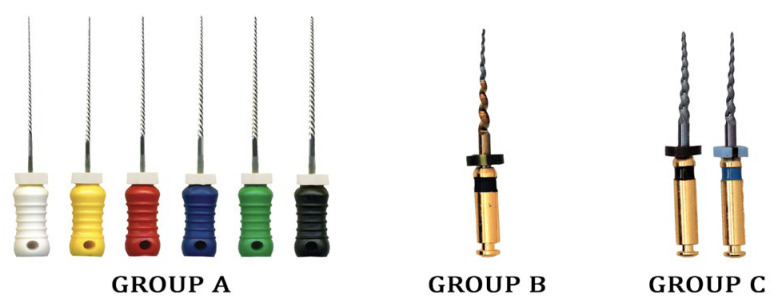
Different file systems used in the present study.

Brand new files were utilized to prepare the canals in the present study. To avoid inter-operator variability, the canals were prepared by a single well-experienced pediatric dentist who has been using these file systems periodically in day-to-day practice. The canal preparation was completed using three different file systems as follows (Figure 2):

Group A file—Hand K-files (Mani Inc., Japan)- Biomechanical preparation was executed using quarter turn and pull motion using stainless steel hand K-files. The instrumentation sequence performed was size 20/0.02 taper, size 25/0.02 taper, size 30/0.02 taper, size 35/0.02 taper and size 40/0.02 taper. For every increase in file size during canal preparation, the samples were irrigated using distilled water. The needle was inserted 2 mm short of working length before irrigation. 

Group B file—Kedo S Plus—A1 Plus file (Kedo Dental, Chennai, India) was used to prepare the canal based on the manufacturer’s instructions. The file was used at 300 rpm and 2 Ncm torque with an electronic endo motor (X-Smart, DENTSPLY India Pvt. Ltd., Delhi, India). The canals were prepared using A1 plus file in pecking motion directed apically until the working length was reached. Once the working length was reached, a brushing motion was performed twice. The samples were irrigated using distilled water before file insertion, once after the pecking motion, once after the brushing motion and finally after the completion of instrumentation. The needle was inserted 2 mm short of working length before irrigation.

Group C—Kedo SG Blue—E1 and U1 files (Kedo Dental., India) were used in sequence to prepare the canal based on the manufacturer’s instructions. The files were used at 300 rpm and 2 Ncm torque with an electronic endo motor (X-Smart, DENTSPLY India Pvt. Ltd., Delhi, India). The canals were prepared using E1 File followed by the U1 file in a pecking motion directed apically until the working length was reached. Once the working length was reached, the brushing motion was performed twice using the U1 file. The samples were irrigated using distilled water before file insertion, once after the pecking motion, once after the brushing motion and finally after the completion of instrumentation. The needle was inserted 2 mm short of working length before irrigation.

Complete irrigation for all the groups was executed using a total of 8 mL of distilled water. This is executed to maintain standardization with the debris amount from the file system. Usage of any canal lubricants such as sodium hypochlorite and ethylenediamine tetraacetic acid were avoided during biomechanical preparation.

### 2.6. Quantifying the Debris

After the completion of biomechanical preparation, the root tips were rinsed (within the Eppendorf tube) with 1 mL of distilled water. A total of 9 mL of distilled water was used per tooth for standardization of the amount of irrigant usage (8 mL during biomechanical preparation and 1 mL for final rinse). The Eppendorf tubes were incubated at 70 °C for 5 days to evaporate the 9 mL of distilled water per tube and sediments were collected at the bottom of the tube. After completion of the incubation period, debris collected from the tube was weighed in milligrams. Three consecutive measurements were completed, and its mean value was noted. The difference between the pre-weight and post-weight of the Eppendorf tube was considered as the weight of the apical debris in milligrams, i.e., Total apical extrusion of debris = Post-weighed Eppendorf tube (−) Pre-weighed Eppendorf tube

#### Statistical Analysis

The collected data were tabulated in an MS Excel sheet. Statistical analysis was performed using the Statistical Package of Social Sciences, version 22.0 (SPSS Inc., Chicago, IL, USA). The distribution of the data was assessed for normality using the Shapiro-Wilk test. The test resulted in a W statistic of 0.965 and a *p*-value of 0.163, indicating that the data is normally distributed. Data collected were analyzed statistically using the one-way analysis of variance (Inter-group comparison) and Tukey’s post hoc analysis (Intra-group). The level of statistical significance was set at *p* < 0.05.

## 3. Results

The maximum extrusion of apical debris was noticed with the Hand K-file system (1.9963 ± 0.12 mg). The least debris was noticed in the Kedo S Plus file system (0.6561 ± 0.06 mg). While Kedo SG Blue had apical debris of 0.10215 ± 0.04 mg (Table 1).

Tukey’s post hoc analysis reveals that there were highly significant differences in apical extrusion and debris when comparing hand files and rotary files and also between the two rotary files used (*p* < 0.05) (Table 2).

## 4. Discussion

Chemomechanical preparation, being the crucial step in pulpectomy, requires preparation and enlargement of the canal space that can help to fill in the obturating material. The wider apical diameter of primary teeth leads to more apically extruded debris in contrast to permanent teeth which have a narrow apical diameter. Usage of any file system in sequence leads to the accumulation of dentinal chips and shavings, micro-organisms, pulp tags and fragments, necrotic contents and irrigants in the canal. This would inadvertently get disseminated into the periapical tissues, which is thought to play a role in multiple sequelae of complications such as postoperative pain, periapical inflammation, operative flare-up and delayed healing causing endodontic failures [25]. Multiple factors influence the amount of apically extruded debris such as the technique of canal preparation, kinematic of the file system, file design, number of file sequences, apical size of the file, type of irrigant solution, irrigant delivery method and the apical diameter of the tooth of concern [26]. Understanding the canal anatomy and endodontic file systems can help in minimizing apical debris extrusion.

Whilst several methodologies have been followed for the quantification of apically extruded debris, the present in vitro study employed the generally accepted and most frequently cited Myers and Montgomery model [24]. Although this method allows separate quantification of debris and irrigant extrusion, the main drawback would be an absence of periapical pressure provided by the periodontium. Other disadvantages include the sensitivity of the analytical balance used, and exposure to moisture leading to the hydration of debris. The other model by Sungur et al. [27] used floral foam which replicates the periapical tissue, and the main drawback of this model is the absorption of extruded debris by the floral foam. So, we did not make any attempt to simulate the periapical tissue resistance.

To maintain standardization, samples used were single-rooted with straight and single canals which is ensured by taking radiographs in different directions. The working length determined is 1 mm shorter than the apex as the previous research has shown extruded debris is higher when instrumentation is completed till the apex. For irrigation, the amount of irrigant used was also standardized among all the groups with the use of equal amounts of 9 ml distilled water which would avoid any excess use of irrigant that can influence the results. Distilled water does not have crystalline sediments which may influence the outcome. All these factors were standardized to avoid any influence over the results.

Although the paradigm shifts in endodontic instrumentation in primary teeth occurred in the early 2000s [28], the extrusion of apical debris is still inevitable. The design, the metallurgy and the motion of the file system play a critical role in the extrusion of apical debris. Comparing hand and engine-driven systems, hand files showed greater extrusion of apical debris in primary teeth compared to rotary file systems [29,30]. The engine-driven rotary instruments used in rotation motion cause lesser extrusion as they tend to pull it along with their flutes coronally [31,32]. Considering the motion of the file systems, the reciprocating files produce conflicting results with one author concluding more apical extrusion than full sequence rotary files [33], while another author concluded less apical debris extrusion than multi-file rotary system [34]. Based on metallurgy, files made of blue alloy preserve apical constriction better than M-wire alloy causing lesser extrusion of apical debris [35].

Kedo-S rotary systems have been upgrading their file designs for easier and more efficient preparation since 2017. Kedo-SG blue is a third-generation Kedo-S file system. It consists of three NiTi files: D1, E1 and U1. Their cross-section is triangular, with a three-point contact, negative rake angle and non-cutting tip design. They are made of M-wire technology which undergoes a proprietary thermomechanical procedure with a special titanium oxide coating (the reason for the blue color after the heat treatment) [36] which provides superior flexibility for the file. Due to the property of controlled memory of the martensitic phase during the complex heating–cooling treatment, these files have a greater resistance to cyclic fatigue, better cutting efficiency, and canal centering ability and also allow intentional deformation with shape memory only when heated [35,37,38,39,40]. The taper is variably variable (taper varies from 4% to 8% at different sections of the flute length) and has a 16 mm file length with a 12 mm flute, which makes it unique among its competitors in the field of pediatric endodontics. Kedo-S Plus is a fifth generation that consists of a similar design but incorporates two different metallurgies into a single file system. The file has undergone two different stages of heat treatment thereby incorporating the gold and blue technology in the single file. The apical 7 mm is heat treated before and after manufacturing with a special titanium oxide coating (blue color), while the initial 5 mm flute length coronally is heat treated before the manufacturing process (gold color) [40]. This allows a higher flexibility in the apical 7 mm which pertains to the highly curved nature of the ribbon-shaped canals of primary teeth. The coronal portion is slightly flexible causing a slightly rigid nature leading to increased canal preparation near the orifice. This allows easier access and flow of the obturating material into the prepared canal space.

The result of the current study shows that hand K-files had the highest apical extrusion debris and Kedo S-Plus had the least apical extrusion debris. Both the rotary file systems used in the present study showed significantly lower extrusion of apical debris compared to hand files (*p* < 0.05). The result of the current study is similar to other studies performed in primary dentition [22,41,42,43,44,45]. While in permanent dentition, contradicting results exist where Madhusudhana et al. suggested minimal debris extrusion with rotary files [46], while Yeter et al. suggested no difference among the file systems [47]. Rotary file systems follow the crown-down technique where the coronal third of the canal is enlarged first followed by the middle third and apical third [30]. Continuous rotary motion during instrumentation of Kedo-SG blue and Kedo-S plus file systems with engine-driven and balanced force concept helps in debris collection in the canal that acts such as a screw conveyor facilitating its evacuation in coronal direction rather than apical direction [48,49]. Moreover, the patented variably variable taper design would still allow some amount of debris suspension in the canal space that would eventually be pushed coronally. This is contradicting the results of Uygun et al. who concluded that taper design did not influence the debris output [50]. Another reason could be the use of martensitic alloy for the manufacture of these rotary files which allows less debris extrusion compared to conventional austenitic alloy [38]. Hand K-files have a smaller constant taper (0.02%), watch winding or quarter pull motion technique used, leading to the formation of debris that is pushed apically. This is due to the minimal remnant space in the canal that hinders the coronal movement of debris forming an apical piston at the apical third of the canal [20,47]. This piston effect at the apical two-three mm would eventually push the debris periapical due to the pecking push-and-pull motion of the hand file systems [51,52].

Both the rotary file systems did allow some amount of debris extrusion apically. This could be due to the dense core and three-point contact of the Kedo-S file systems that provide minimal space for debris suspension would still allow extrusion to an extent [53]. Furthermore, any instrument that is directed apically within the canal, does allow plunging action leading to extrusion [54]. Compared within the rotary file systems, Kedo-S plus had significantly lower debris extrusion compared to Kedo-SG blue (*p* = 0.000). This could be attributed to the difference in heat treatment protocols during the manufacture of the Kedo-S plus file system. The gold-coated coronal 5 mm of the flutes produced increased preparation of the canal near the orifice that could allow more debris accumulation in the coronal aspect during the crown-down technique. This is assisted by the rotational movement of the file that leads to easier coronal movement of the debris thus significantly reducing the apical debris extrusion. Additionally, the number of instruments used could be an additional factor in the debris output. Kedo-SG blue involved two instruments E1 and U1 during the instrumentation procedure while Kedo-S plus involved only A1 plus file (single file system). This observation was similar to the study completed by Topçuoğlu [30], Vivekanandhan et al. [48], and Ehsani et al. [55]. This result contradicts previously published studies that showed the number of instruments used does not have a direct effect on the debris output [21,50].

The limitation of the current in vitro study is that the assessment method used does not fully reflect the clinical viewpoint. The results of the current study can be applied to an ideal single canal, straight-rooted primary maxillary central incisors with no apical resorption in an in vitro set-up without an apical barrier. Moreover, the extrusion of the debris might be dependent on the different instrument designs, instrumentation techniques and irrigants used. The type of roots chosen for the study also could be a reason for lower levels of debris. Wider canals of incisors would reduce the pumping action of the file during insertion resulting in less debris. Primary molars with narrower canals could extrude more debris due to minimal coronal flaring. The usage of side-vented needles for irrigation could have reduced the extrusion. However, this type of needle was not followed in the present study [55]. However, further investigations based on post-operative flare-ups, post-operative pain, and obturation quality would be necessary to support its clinical significance. Micro-CT or the more recent Nano-CT analysis can also be performed to assess the quality of canal preparation which would provide more laboratory evidence on the extent of debris formed which might correlate clinically. Furthermore, it’s not just the quantity of debris that leads to endodontic failure, but also the virulence level of a specific type of bacterium that is present along with the debris and the antibacterial action of intracanal medicaments and sealers used [56].

## 5. Conclusions

Within the limitations of the study, it was noticed that apical debris collection is an inevitable outcome of canal instrumentation. Among the file systems compared, rotary files had lesser extrusion when compared to hand files. Among the rotary files, Kedo S plus showed normal extrusion compared to SG Blue. Further in-vivo studies on post-operative pain and flare-ups are required to evaluate the clinical application of using the file systems.

## Figures and Tables

**Table 1:** Mean weight of apically extruded debris among the three groups of endodontic file system.

Groups	*n*	Weight of Apically Extruded Debris ± SD (In Milligrams)
Group A (Hand K-file)	20	1.9963 ± 0.12
Group B (Kedo S plus file)	20	0.6561 ± 0.06
Group C (Kedo SG Blue file)	20	0.10215 ± 0.04

*p* < 0.05. SD: Standard deviation.

**Table 2:** Comparison of difference in extrusion of apical debris caused by different file systems based on Turkey’s post hoc test.

Groups	Comparison Groups	Significance
Group A (Hand K-file)	Group B (Kedo S plus file)	0
	Group C (Kedo SG Blue file)	0
Group B (Kedo S plus file)	Group A (Hand K-file)	0
	Group C (Kedo SG Blue file)	0
Group C (Kedo SG Blue file)	Group A (Hand K-file)	0
	Group B (Kedo S plus file)	0

## Data Availability

No data availability.
